# Supplementary material for: The Effects of Gluconacin on Bacterial Tomato Pathogens and Protection against Xanthomonas perforans, the Causal Agent of Bacterial Spot Disease
Source: Plants (Basel). 2023 Sep 8;12(18):3208. doi: 10.3390/plants12183208 (PMC10535834; doi:10.3390/plants12183208)
Supplement: Supplementary file 1 [file plants-12-03208-s001.zip › plants-2531083-supplementary.pdf]

The effect of gluconacin on bacterial tomato pathogens and on protecting the plant against *Xanthomonas perforans*, the causal agent of bacterial spot disease

Elizabeth Teixeira de Almeida Ramos [1,5], Fábio Lopes Olivares [2], Letícia Oliveira da Rocha [2], Rogério Freire da Silva [3], Margarida Goréte Ferreira do Carmo [1], Maria Teresa Gomes Lopes [4], Carlos Henrique Salvino Gadelha Meneses [3], Marcia Soares Vidal [5] and José Ivo Baldani [5,*]

SUPPORTING INFORMATION

Additional Supporting Information may be found in the online version of this article:

Table S1. The minimum inhibitory concentration of gluconacin for phytopathogenic bacteria of sugarcane and tomato.

Bacterial species	MIC ($\mu g.\mu L^{-1}$)
**Sugarcane phytopathogenic**	
X. vasicola pv. *vasculorum*	0.25 ±0.01[c]
P. syringae pv. *syringae*	1.0 ±0.04[a]
X. axonopodis pv. *vasculorum*	1.0 ±0.07[a]
X. albilineans	0.25 ±0.01[c]
A. venae subsp. *avenae*	1.0 ±0.05[a]
**Tomato phytopathogenic**	
P. syringae pv. *tomato*	1.0 ±0.09[a]
R. solanacearum	0.5 ±0.04[b]
X. perforans	1.0 ±0.03[a]

Standard deviation (±) was calculated from the results of the three replicates.
